# A Tale of Two Hypersecreting Adrenal Neoplasms in the Heartland of COVID-19 Pandemic, Lombardy, Italy

**DOI:** 10.1155/2022/1539203

**Published:** 2022-02-18

**Authors:** Benedetta Zampetti, Roberto Attanasio, Emanuela Carioni, Daniela Dallabonzana, Iuliana Pauna, Marco Boniardi, Renato Cozzi

**Affiliations:** ^1^ASST Grande Ospedale Metropolitano Niguarda, Endocrinology Department, Milan, Italy; ^2^Scientific Committee Associazione Medici Endocrinologi, International Chapter of Clinical Endocrinology, Milan, Italy; ^3^ASST Grande Ospedale Metropolitano Niguarda, General Oncologic and Mini-invasive Surgery Department, Milan, Italy

## Abstract

In this study, we report the management, in Lombardy, Italy, of one patient with Cushing's syndrome due to adrenal adenoma and another one with pheochromocytoma, whose surgeries were deferred owing to the COVID-19 pandemic.

## 1. Introduction

Surgery is the mainstay of treatment for hypersecreting adrenal lesions, whenever clinically and technically feasible [1, 2].

COVID-19 pandemic obliged physicians to find out alternative options to classical pathways, in order to lower viral spread and related dangers as well as to cope with redeployment of personnel and resources.

We describe here two cases in whom surgery for adrenal Cushing's syndrome (CS) and pheochromocytoma (PHEO) could not be performed as usual and was deferred due to the unavailability of surgical facilities.

## 2. Case 1

A 69-year-old woman was evaluated for CS.

Her history included arterial hypertension for 10 years, hyperlipidemia, and euthyroid multinodular goiter.

In 2017, type 2 diabetes mellitus was diagnosed, and a 35 mm right adrenal mass was incidentally found at US at another hospital. No endocrine work-up was performed. Due to high density (40 HU) of the lesion, a ^18^F-FDG-PET scan was performed with negative results. The size of the lesion was unchanged at CT follow-up in the following two years.

In December 2019, clinical picture worsened with progressive loss of independence and self-care ability up to walking impairment. Physical examination was suggestive of hypercortisolism (central obesity, muscle atrophy, moon facies, buffalo hump, and leg ulcers). Endocrine work-up showed ACTH-independent hypercortisolism: UFC was 214 nM/24 h (by LC-MS/MS, nv <168), serum cortisol after overnight 1 mg dexamethasone test was 240 *μ*g/L (nv <18), and ACTH 1 ng/L (normal value: 7–63 ng/L). Urinary metanephrines were within the normal range. CT scan confirmed a 35 mm right adrenal mass.

In July 2020, during the full-blown pandemic period, glucometabolic derangement required hospitalization in our hospital. Nasal swab was negative for SARS-CoV-2, fasting glucose was 300 mg/dL, HbA1c 12.5%, eGFR 34 mL/min/1.73 m^2^, AST 39 U/L, ALT 60 U/L, and gamma-GT 608 U/L. Abdominal CT did not show additional lesions beyond the known 36 × 33 mm right adrenal nodule; in particular, the liver and biliary tree were normal. Screening for complications of CS ruled out vertebral fractures and disclosed hypertrophic cardiomyopathy with outflow obstruction.

The whole picture was so severe that she was not considered suitable for surgery that was anyway inaccessible due to pandemic. The impairment in the liver function test (LFT) was attributed to steroid hepatitis.

Initial daily treatment included bisoprolol 5 mg, perindopril 10 mg, amlodipine 10 mg, doxazosin 4 mg, furosemide 50 mg, spironolactone 37 mg, atorvastatin 20 mg, and acetylsalicylic acid 100 mg. Antidiabetic treatment was uptitrated with insulin (total daily dose: 84 U; degludec, 38 U, fast-acting aspart, 14 + 18 + 14 U), dapagliflozin 10 mg/day, and long-acting exenatide 2 mg/week.

Medical treatment with ketoconazole (KCZ) was then cautiously administered, starting with 200 mg/day and then uptitrated to 400 mg/day. The choice of KCZ was due to transient unavailability of metyrapone (osilodrostat was not yet marketed). The patient complained nausea, and KCZ dose was downtitrated to 200 and 400 mg every other day. UFC values progressively decreased to 38 *μ*g/24 h (nv 35–137) as well as transaminase and gamma-GT values, and cortisone acetate 12.5 mg/day was added. This block-and-replace schedule was well tolerated, clinical picture improved, and insulin dose was progressively reduced to 70 U/day due to the continuous occurrence of hypoglycemic episodes.

She was discharged home clinically improved, with the indication to weekly control of LFT, insulin dose tapering according to glucose automonitoring and medical advice, and quick glucocorticoid dose increase in case of fever or stress. Adrenal resection should have been planned as soon as possible in relation to clinical improvement and pandemic evolution.

After one month, she was hospitalized in another ward owing to syncope for acute adrenal failure due to symptomatic febrile urinary infection without a timely quick increase of glucocorticoid dosage. The daily block-and-replace schedule was adjusted to KCZ 400 mg plus cortisone acetate 25 mg. This treatment induced a progressive amelioration of clinical picture, including healing of leg ulcers, mood improvement, and restoring of the ability to walk. LFT normalized, antihypertensive drug dosage was reduced, HbA1c dropped to 6.2%, and insulin dose was reduced to 26 U/day owing to frequent hypoglycemic episodes. UFC was now normal (ranging 31–52 *μ*g/24 h).

Due to the persistence of the COVID-19 pandemic in our region and the unavailability of any surgical facilities for not urgent surgeries, despite our pressing on surgeon and hospital management, the patient is still scheduled for adrenal resection. She is carrying on KCZ plus cortisone acetate therapy at fixed dose, with the advice to quickly increasing the steroid dose as soon as any suspect of impending infection occurs. A progressive improvement in clinical and biochemical picture has been observed on this medical treatment that is aimed to keep a good control of arterial pressure and a fair control of the glucose metabolism while avoiding any hypoglycemia.

In July 2021, abdomen CT scan did not show any change in tumor size; the values of UFC and ACTH were 35 *μ*g/24 h and 1.5 ng/L, respectively, on a block-and-replace treatment (KCZ 400 mg plus cortisone acetate 25 mg/day).

## 3. Case 2

An 83-year-old woman was evaluated for cardiogenic shock.

Her history was remarkable for hyperlipidemia, gastroesophageal reflux, right breast cancer (in 2000 with negative annual follow-up), and surgically treated autonomous thyroid adenoma (in 1980). In addition, she had undergone cholecystectomy in 2018 that was complicated by severe hypertensive crisis with acute heart failure.

In September 2019, abdominal plastic surgery was complicated by severe hypertensive crisis with acute pulmonary edema, cardiogenic shock, and acute renal failure. She was thus transferred to ICU for suspected acute coronary syndrome. Multiple noncritical stenoses were disclosed at coronary angiography and reverse Takotsubo at ventriculography. Chest CT disclosed a left adrenal lesion consistent with PHEO. Conservative therapies (ramipril, lercanidipine, atenolol, and furosemide) induced recovery of systolic function (ejection fraction increased from 5% to 62%).

Endocrine work-up confirmed PHEO: urinary adrenaline was 34 *μ*g/24 h (nv <22), noradrenaline 81 *μ*g/24 h (nv <85), metanephrines 1932 *μ*g/24 h (nv <297), and normetanephrines 2009 *μ*g/24 h (nv <354). At retrospective inquiring, the patient reported palpitations, tremors, diaphoresis, headaches, anxiety, and panic attacks. Fasting plasma glucose was 79 mg/dL and HbA1c 5.2%.

Abdominal CT and MRI confirmed a 45 × 31 mm left adrenal lesion, with irregular margins and inhomogeneous contrast enhancement, whose density was 30 HU, without other remarkable lesions.

The planning of adrenal resection was postponed due to an adrenal hematoma developed while on LMWE treatment. Pressure control was maintained with doxazosin (in addition to ongoing treatment) titrated up to 8 mg bid. In January 2020, CT showed hematoma reabsorption, but while waiting for scheduled surgery, there was the outbreak of pandemic. The patient was monitored throughout Spring and Summer with frequent telemedicine controls that confirmed optimal pressure control, without any side effect of antihypertensive treatment.

In October 2020, nasal swab was negative for SARS-CoV-2, and she underwent laparoscopic left adrenalectomy, taking advantage of a transient decrease of pandemic burden.

Postoperative recovery was uneventful, no hypotension occurred, and the patient was discharged home on atenolol and doxazosin that were quickly tapered and finally discontinued within one month. Histologic examination confirmed the diagnosis of PHEO: the lesion infiltrated the perivisceral adipose tissue due to the previous hematoma, but there was no angioinvasion, Ki-67 was <1%, and PASS score was 7.

One month later, urinary metanephrines were normalized, namely, 93 and 282 *μ*g/24 h for metanephrine and normetanephrine, respectively, and remained normal at the following control.

A recent CT did not disclose any tumor remnant or relapse.

## 4. Discussion

CS is a life-threatening disease, with vascular disease and infections representing the most common cause of death, mainly in elderly patients. Both diagnosis and management of CS might be cumbersome even in ordinary times, mostly in patients with a cyclic disease. The diagnosis in patient 1 was straightforward but the management was not, due to the COVID-19 pandemic. Surgery is the first-line treatment for CS, regardless of its etiology [1], but in some extremely frail patients, it should be postponed even in ordinary times, in order to ameliorate clinical picture with steroidogenesis inhibitors according to clinical status. Furthermore, surgery should be considered with caution during the pandemic because cortisol hypersecretion may lead to a lessening of immune reaction. In addition, following an issue of hospital management, only urgent cases should be admitted to surgery unit due to the shortage of intensive care units and healthcare personnel.

We followed thus successfully the recommendations of the European Society of Endocrinology [3] that points to surgery only in patients who cannot be controlled with medical therapy or in those requiring biopsy or resection because of possible cancer.

The infective risk is indeed increased by defective innate and adaptive immune response due to chronic glucocorticoid exposure [4]. The increased susceptibility to infection is further increased by visceral obesity, hypertension, and diabetes that are characteristics of the disease and hugely heighten the risk of death. Furthermore, CS patients have high risk of thromboembolic events due to many alterations of coagulation and fibrinolysis that in turn worsen the prognosis in the case of COVID-19 infection. We opted thus for drug treatment to get control of the disease while waiting for more propitious times for a surgical approach.

Among the different steroidogenesis inhibitors, we had to choose KCZ because osilodrostat was not marketed yet, and there was a temporary shortage of metyrapone supply. Keeping in mind the liver toxicity of KCZ, reportedly occurring in 3–19% of treated patients [5], this choice might have been regarded as hazardous in a patient with severe derangement of LFT. Anyway, the severity of clinical picture prompted us to start treatment with a tight monitoring. Treatment obtained actually a quick and progressive improvement in clinical and biochemical picture. No escape was observed, on the contrary adrenal failure developed prompting the use of a block-and-replace schedule. This option has proved effective beyond all our expectations.

In patient 2, the diagnosis of PHEO was neglected for a long time, until catastrophic acute worsening of cardiac function occurred that required intensive treatment.

According to Endocrine Society guidelines, the treatment for PHEO is surgical adrenalectomy after a brief period of medical preparation (7–14 days) with alpha-adrenergic receptor blockade to minimize perioperative complications [2].

The unusual circumstances created by the pandemic necessitated a more prolonged preoperative period of medical management, as already described in other 4 cases recently reported [6, 7]. At last, the adrenal tumor was successfully resected after a prolonged pharmacologic control of blood pressure, and the patient is doing well off antihypertensive treatment.

This effort of progressive continuous adjustment should always take into account both the epidemiological environment with its constraints and the individual clinical picture.

## Abbreviations

ACTH: Adrenocorticotropic hormone
ALT: Alanine transaminase
AST: Aspartate transaminase
COVID-19: Corona-virus disease 2019
CS: Cushing's syndrome
CT: Computerized tomography
eGFR: Estimated glomerular filtration rate
gamma-GT: Gamma-glutamyl-transferase
HbA1c: Glycated hemoglobin
HU: Hounsfield units
KCZ: Ketoconazole
LC-MS/MS: Liquid chromatography-tandem mass spectrometry
LFT: Liver function test
LMWE: Low molecular weight heparin
MRI: Magnetic resonance imaging
Nv: Normal values
PASS: Pheochromocytoma of the Adrenal Gland Scaled Score
PET: Positron emission tomography
PHEO: Pheochromocytoma
UFC: Urinary free cortisol
US: Ultrasonography.
